# Novel Approaches to Memory and Aging: The Editorial

**DOI:** 10.3390/brainsci13030518

**Published:** 2023-03-21

**Authors:** Caterina Padulo, Beth Fairfield

**Affiliations:** Department of Humanities, University of Naples, Federico II, 80133 Naples, Italy

Heightened average life expectancy, which is increasing the number of older adults destined to live alone in the future, is forcing society to acknowledge the strong positive correlation between health costs and age. Accordingly, health systems, and welfare in general, must be reformed to focus on the prevention of cognitive and physical impairment and social isolation, as well as the promotion of successful aging in innovative ways. Indeed, this is essential because aging is a normal physiological process that can develop without the appearance of concurrent diseases; however, even in the absence of dementia, older people frequently suffer from memory loss, accelerated cognitive decline, and modifications in memory abilities that significantly affect their quality of life [1]. Therefore, research is crucial for better understanding the aging brain, as well as behaviors linked to these changes. What is more, there is an urgent need to understand how aging affects cognition, especially memory, because the ability to learn new and retrieve previously learned information is essential for successful aging and to adapt to changes in the environment [2,3].

To this end, we invited authors with expertise in memory and aging to submit original research, review articles, and commentaries to the present Special Issue, especially papers focusing on novel approaches for understanding the mechanisms and processes underlying age-related memory performance. Our authors contributed 13 thought-provoking studies on different aspects of memory functions, including 11 original articles, 1 perspective paper, and 1 review that aimed to further knowledge on aging by investigating different aspects of underlying age-related memory modifications and by drawing upon diverse methodologies.

One group of studies investigated the possible contributions of specific stimuli, training protocols, and recognized effects to the enhancement of memory retrieval and/or recall. In particular, across two experiments, Polden and Crawford [4] reported no memory retrieval enhancement effects for the “saccade-induced retrieval effect” (SIRE) in both normal and pathological aging, highlighting the SIRE effect’s apparent lack of strength. On the contrary, both curiosity [5] and affective information [6] may affect information processing by improving memory for trivia answers and incidental memory for unrelated faces, simplifying information processing by both prioritizing positive information and de-prioritizing negative information, respectively. These outcomes suggest that in designing and implementing prevention and promotion protocols, researchers need to consider motivational factors, such as curiosity, and focus on the presentation of positive and negative information in competition instead of limiting attention to isolated positive and/or negative manipulations. Additionally, incidental memory seems to benefit from wakeful rest (i.e., a brief period of about 10 min of minimal stimulation while individuals are awake) across age groups, supporting its potential suitability in older adults’ consolidation mechanisms [7].

Čepukaitytė et al.’s study [8] adopted a rigorous experimental control and novel continuous-report tasks to evaluate both short- and long-term memories and the relationship between the two memory systems and age-linked changes. Overall, they found that aging impairs both short- and long-term memory performance but does not alter the significant relationship between these two memory functions.

Vannucci et al. [9] examined the age-linked dimensions of well-being to investigate whether interactions predicted the phenomenological properties of semantic self-images, which are representations in autobiographical memory that aid retrieval and memory for autobiographical events. They found a strong association between well-being and semantic self-images, especially for positive and negative affects and life satisfaction. Moreover, self-statements accessibility seemed to reflect the order of statement generation. Similarly, Harris et al. [10] assessed the role of teaching elaborative reminiscing in supporting autobiographical memory and relationships in aged care services to improve dialogue between staff and residents. They found that elaborative reminiscing techniques were successful in improving the recall of episodic and semantic autobiographical memory details, underlining the importance of autobiographical memory across lifespan and in conditions of cognitive need.

In the area of cognitive intervention for adults, Strickland-Hughes and West [11] examined the recall, memory self-regulation, and near-transfer effects of a revised brief version of the Everyday Memory Clinic (EMC) [12], a multifactorial memory training program for adults over the age of 50. The authors found that after a single week of training, participants showed improvements in all three dimensions.

Tuena et al. [13] examined virtual reality as a preferential candidate for spatial memory rehabilitation based on the observation that declines in body-related information are often observed in aging, and are thought to contribute to impairments in navigation, memory, and space perception. According to the authors, existing virtual neurorehabilitation programs for aging and neurodegenerative diseases need to be ameliorated, and they propose innovative rehabilitative solutions in aging for spatial memory and navigation by presenting interesting theoretical and empirical findings.

Switching from physiological to pathological aging, Putcha et al. [14] examined California Verbal Learning Test (CVLT-II-SF) performance in amyloid-, tau-, and neurodegeneration-positive participants with atypical non-amnestic and amnestic variants of Alzheimer’s Disease (AD). They found that both non-amnestic and amnestic patients showed weak recognition memory. However, while non-amnestic AD patients showed difficulties in encoding, the amnestic AD group showed poor recognition memory irrespective of encoding capacity. In addition, the pattern of cortical atrophy was differently distributed between the memory network related to encoding and that related to recognition memory.

Gómez-Ramírez et al. [15] applied machine learning from Magnetic Resonance Imaging (MRI) brain segmentation and cortical parcellation to assess age-related brain changes in normal brain aging. They found brain-to-intracranial-volume ratio to be the most significant aspect in predicting age, followed by hippocampal volume, and discovered cortical thickness in temporal and parietal lobes to be more predictive than frontal and occipital lobes. In addition, McDonough et al. [16] used structural and functional resting-state MRI to discover that younger adults with a parent with dementia show greater resting mean activity and smaller volume in the left hippocampus compared with those without, suggesting that having a parent with AD or a related dementia is associated with early aberrations in brain function and structure, possibly increasing the risk for a diagnosis of dementia in old age.

Finally, Passarello et al.’s review [17] discussed motor imagery as a key factor for healthy aging, providing an interesting overview of the scientific literature in the last decade focused on age-related changes in motor imagery skills. The authors emphasized the challenges and potentialities of motor imagery skills in therapeutic and rehabilitation contexts, as well as providing evidence from neuroimaging studies that revealed that the hyperactivation of sensorimotor and attentional networks seems to represent a compensatory mechanism for one individual, while also being a sign of cognitive decline for someone else.

In summary, the articles collected in this Special Issue are representative of ongoing research in the active area of age-related memory performance and change, providing stimulating reading and important findings and suggestions for interested readers from clinical and research backgrounds.

## References

[1] Parikh P.K., Troyer A.K., Maione A.M., Murphy K.J. (2016). The impact of memory change on daily life in normal aging and mild cognitive impairment. Gerontologist.

[2] Padulo C., Mammarella N., Brancucci A., Altamura M., Fairfield B. (2020). The effects of music on spatial reasoning. Psychol. Res..

[3] Padulo C., Mammarella N., Brancucci A., Fairfield B. (2021). Memory for item–location bindings is enhanced in older adults with appetitive motivationally laden pictures. Psychol. Res..

[4] Polden M., Crawford T.J. (2022). On the Effect of Bilateral Eye Movements on Memory Retrieval in Ageing and Dementia. Brain Sci..

[5] Padulo C., Marascia E., Conte N., Passarello N., Mandolesi L., Fairfield B. (2022). Curiosity Killed the Cat but Not Memory: Enhanced Performance in High-Curiosity States. Brain Sci..

[6] Fairfield B., Padulo C., Bortolotti A., Perfetti B., Mammarella N., Balsamo M. (2022). Do Older and Younger Adults Prefer the Positive or Avoid the Negative?. Brain Sci..

[7] Millar P.R., Balota D.A. (2022). Wakeful Rest Benefits Recall, but Not Recognition, of Incidentally Encoded Memory Stimuli in Younger and Older Adults. Brain Sci..

[8] Čepukaitytė G., Thom J.L., Kallmayer M., Nobre A.C., Zokaei N. (2023). The relationship between short-and long-term memory is preserved across the age range. Brain Sci..

[9] Vannucci M., Chiorri C., Pelagatti C., Favilli L. (2022). Semantic Self-Images and Well-Being in Young and Older Adults: Does the Accessibility Matter?. Brain Sci..

[10] Harris C.B., Van Bergen P., Strutt P.A., Picard G.K., Harris S.A., Brookman R., Nelson K. (2022). Teaching elaborative reminiscing to support autobiographical memory and relationships in residential and community aged care services. Brain Sci..

[11] Strickland-Hughes C.M., West R.L. (2022). Brief Strategy Training in Aging: Near Transfer Effects and Mediation of Gains by Improved Self-Regulation. Brain Sci..

[12] West R.L., Bagwell D.K., Dark-Freudeman A. (2008). Self-efficacy and memory aging: The impact of a memory intervention based on self-efficacy. Aging Neuropsychol. Cogn..

[13] Tuena C., Serino S., Pedroli E., Stramba-Badiale M., Riva G., Repetto C. (2021). Building embodied spaces for spatial memory neurorehabilitation with virtual reality in normal and pathological aging. Brain Sci..

[14] Putcha D., Carvalho N., Dev S., McGinnis S.M., Dickerson B.C., Wong B. (2022). Verbal Encoding Deficits Impact Recognition Memory in Atypical “Non-Amnestic” Alzheimer’s Disease. Brain Sci..

[15] Gómez-Ramírez J., Fernández-Blázquez M.A., González-Rosa J.J. (2022). Prediction of chronological age in healthy elderly subjects with machine learning from mri brain segmentation and cortical parcellation. Brain Sci..

[16] McDonough I.M., Mayhugh C., Moore M.K., Brasfield M.B., Letang S.K., Madan C.R., Allen R.S. (2022). Young Adults with a Parent with Dementia Show Early Abnormalities in Brain Activity and Brain Volume in the Hippocampus: A Matched Case-Control Study. Brain Sci..

[17] Passarello N., Liparoti M., Padulo C., Sorrentino P., Alivernini F., Fairfield B., Lucidi F., Mandolesi L. (2022). Motor Imagery as a Key Factor for Healthy Ageing: A Review of New Insights and Techniques. Brain Sci..

